# Chinese Consumers' E-Learning Satisfaction and Continuance Purchase Intention on Paid Online Python Course

**DOI:** 10.3389/fpsyg.2022.849627

**Published:** 2022-06-13

**Authors:** Jingjing Zhang, Long She, Dongyuan Wang, Ali Shafiq

**Affiliations:** ^1^Faculty of Business and Law, Taylor's University, Subang Jaya, Malaysia; ^2^Faculty of Business, Design and Arts, Swinburne University of Technology, Kuching, Malaysia; ^3^Department of International Economics and Trade, Wuhan University of Technology, Wuhan, China; ^4^Faculty of Business and Law, School of Marketing and Management, Coventry University, Coventry, United Kingdom

**Keywords:** continuance purchase intention, satisfaction, e-word of mouth, e-learning, self-efficacy, Python course

## Abstract

With the development of internet technology, e-learning has become an essential part of the modern education system. However, the e-learning market faces enormous competition. Consumers' continuance purchase intention has become a vital factor in the success of e-learning courses. Thus, factors that influence consumers' continuance purchase intention should be examined in the e-learning market. However, little research has focused on identifying the continuance purchase intention of an e-learning course. Based on the information system continuity model ISC), this paper develops a research model to investigate the factors influencing satisfaction and continuance purchase intention in e-learning. A cross-sectional, questionnaire-based research design was used in this study. We collected data from consumers who had enrolled in paid online Python courses. In total, 508 paid online Python course users completed the online survey. SmartPLS software was used for data analysis. The results demonstrated that perceived course quality, service quality, convenience, and usefulness significantly affect consumers' satisfaction with the experience course. Moreover, the findings show that satisfaction, self-efficacy, and e-word of mouth (e-WOM) determine the consumers' continuance purchase intention of the reminder course. This study also found that satisfaction mediates the effects of experience courses on consumers' continuance purchase intention of the online Python course. The implications for theory and practice and future research directions are discussed.

## Introduction

E-learning is a rapidly emerging trend in the 21st century (Haythornthwaite, 2015; Son, 2019; Siddiquei and Khalid, 2022). Regardless of the challenges and difficulties companies might experience in this shift, industrial facts and figures show that the rise in the e-learning market is inevitable (Kimiloglu et al., 2017; Rajasekaran et al., 2022). According to Global Industry Analysts Inc. (2021), the global e-learning market is expected to reach $457.8 billion by 2026. Over the years, e-learning has become an essential part of the modern education system. More and more training organizations are embracing e-learning to upgrade their education systems and improve competitiveness.

A learning Python course^1^ has become incredibly popular, as it offers useful skills for one's daily work (Coursera, 2021). With the advancement in the digital age and increasing demand for data analysts, possessing Python skills has become a competitive advantage for better employment and salary (Zhang, 2017). In China, the annual salary of a data analyst is 20–30% higher than that of other comparable positions (51CTO, 2021).

A short Python course is easy for an individual to learn since it does not require a background in intensive computer skills. Consequently, more and more Chinese have started learning Python; it has become so popular that some provinces have even made Python a compulsory course in elementary schools (Jiemian, 2017; Aisoutu, 2022). Learning Python in face-to-face classes was not popular in China, despite its popularity. Many cities did not offer a face-to-face Python course simply because people's busy work schedules disallowed them from enrolling in such courses. An example of such a case is tour guides who spend most of their time leading tour groups, disabling them from sitting for such courses (Liu et al., 2020). Therefore, an online Python course has been introduced to reduce time and travel limitations.

Consumer satisfaction is a critical factor in the success of any user-oriented service. It is also true for the successful implementation of e-learning Python in a fiercely competitive market. In general, an online Python course can be divided into two parts: the experience course (which may include two or three modules of the whole course) and the remainder of the course (Sohu, 2020; Thefuturesphere, 2022). The experience course affects consumer satisfaction, which decides if consumers continue to purchase the remainder of the course. According to the Information System Continuance Model (ISC), satisfaction is a vital factor that determines consumers' continuance intention (Hsu et al., 2014; Akdim et al., 2022). The same was witnessed for Python: once consumers were satisfied with the experience course, they continued to the remainder of the course. Consumer satisfaction is a key point that should be considered when studying consumers' continuance intention. Although current research has identified factors that influence consumers' satisfaction in e-learning, there is a lack of empirical research focusing on the specific context of e-learning Python and the factors that affect consumer satisfaction in this context.

The ISC model can be explained in two stages: (1) the satisfaction stage and (2) the consumers' continuance intention stage (Bhattacherjee, 2001). For the purpose of this study, these stages can be referred to as (1) the satisfaction of experiencing the Python course and (2) continuance with the remainder of the course. Previous research has focused on consumers' continuance intention toward using e-learning (Chow and Shi, 2014; Rodríguez-Ardura and Meseguer-Artola, 2016; Suzianti and Paramadini, 2021). However, we do not have sufficient studies focusing on consumers' continuance purchase intention. Since consumers' purchase intention is related to the success of a course or an organization (Peña-García et al., 2020), it is necessary to investigate the factors affecting consumers' continuance purchase intention of the e-learning course. Such a detailed analysis of the two stages of an e-course will produce a clear understanding of consumers' cognitive and behavioral processes at different stages of the courses, which will help the course providers improve the course and adjust their marketing strategy.

## Theoretical Development and Hypotheses

### Information System Continuance Model

The information system continuance model (ISC) describes the use of information systems after the initial acceptance stage in which a consumer decides to start using a product (Bhattacherjee, 2001). Knowledge about the long-term usage of an information system may be more important than knowing only about its initial acceptance. The system provider benefits most from a sustained relationship (Bhattacherjee, 2001). The ISC model measures four constructs: continuance intention, satisfaction, perceived usefulness, and confirmation (Bhattacherjee, 2001; Cai et al., 2022). It also explains the acceptance-discontinuance anomaly, which describes the phenomenon when consumers start using a system and later discontinue its use (Budner et al., 2017).

**Figure 1:**
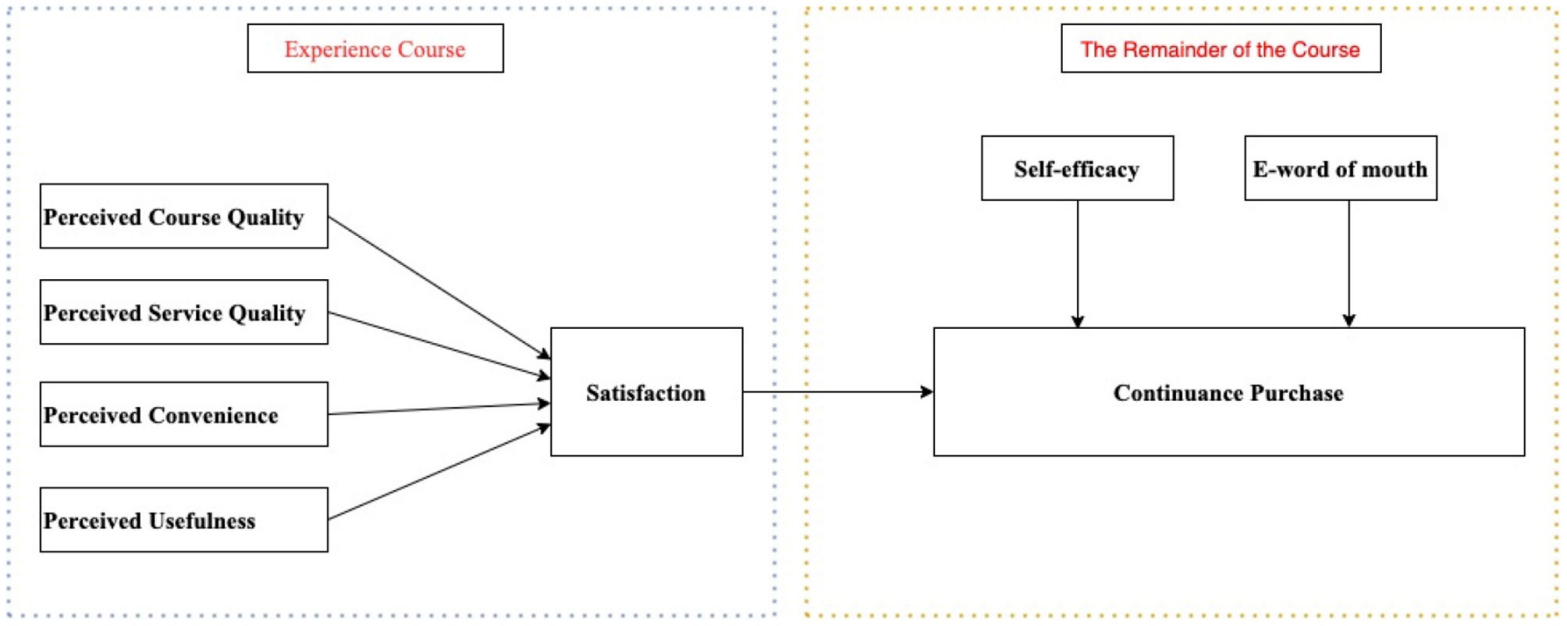
Research model.

The ISC model has been extended and widely adopted in the context of e-learning (Hong et al., 2017; Franque et al., 2020). It is used to explain consumers' satisfaction and continuance intention with the e-learning process (Nugroho et al., 2019). An underlying model is mainly used to predict consumers' satisfaction and continuance intention by exploring and identifying the critical factors in the e-learning process (Yassine et al., 2017). The core of the ISC model's theoretical attributes is to provide a robust and concise framework for continuous use; only three variables (satisfaction, perceived usefulness, and confirmation) are needed to provide the core theoretical explanation behind continuous decision-making (Bøe, 2018). It is convenient and helpful for researchers to develop a new model based on a simple and powerful continuance model. Thus, this research uses the ISC model to identify the factors that influence consumers' satisfaction and continuance purchase intention of the e-learning Python courses. This may also extend the ISC model and be a useful model of e-learning in the new area. Figure 1 presents the research model for this study.

### Perceived Course Quality and Satisfaction

Course quality is the quality of e-learning system outputs of learning resources (Li et al., 2012). The reality of the competitiveness of the e-learning market makes course quality for online education even more crucial (Pham et al., 2022). Additionally, the quality of online courses is especially critical for learners to consider whether they intend to continue using the online platform once they finish the current courses (Liu et al., 2010). Therefore, for e-learning institutions, it is critical to evaluate, verify, and convey the quality of their courses to potential consumers (Zimmerman et al., 2020). Moreover, course quality positively contributed to learners' satisfaction, which led to their continuance intention (Joo and Choi, 2016; Cheng, 2020). In this study, perceived course quality refers to consumers' perception of Python course quality, specifically, the perceived quality of their experience of the course.

Satisfaction reflects consumers' response when they compare the expectation with the experience after utilizing a product or service offered by a company (Chen et al., 2012; Tj and Tanuraharjo, 2020). It usually happens when the consumer compares their perceptions before and after purchasing a product or service (Chen et al., 2012). Consumer satisfaction is important, and businesses must maintain it to survive and succeed in competition (Tj and Tanuraharjo, 2020). In recent years, satisfaction has been widely considered in e-learning (Erfannia et al., 2022; Ung et al., 2022). Satisfaction with an online course is a variable that involves a self-perceived range of fulfillment linked to the individual's wish to learn and is an indicator of a positive evaluation of a learning experience (Dal Santo et al., 2022). Besides, satisfaction has been regarded as a determinant of the success of e-learning systems (Sugandini and Istanto, 2022).

Research has proved that course quality significantly contributes to consumer satisfaction (Li et al., 2012; Cheng, 2020). For example, Erfannia et al. (2022) explained that better support of e-learning course quality resulted in more fabulous students' satisfaction at Zahedan University of Medical Sciences. Cheng (2020) indicated that course quality (content quality and design quality) positively contributed to students' satisfaction with the cloud-based e-learning system. Therefore, based on the above, Hypothesis 1 is proposed.

**H1**. Perceived course quality positively affects satisfaction.

### Perceived Service Quality and Satisfaction

In general, e-learning service quality includes system quality, instructors, and course materials (Tj and Tanuraharjo, 2020). Many researchers have examined service quality as another important antecedent to consumer satisfaction in the context of e-learning (Almahamid and Rub, 2011; Pham et al., 2018; Bae and Shin, 2020). Institutions have focused their attention on improving e-learning service quality, as successful adoption of e-learning can be caused by service quality (Sugandini and Istanto, 2022). Poor service support could lead to lost consumers and even lost sales (Achmadi and Siregar, 2021). Thus, for e-learning institutions to survive in an increasingly competitive e-learning environment, it is clear that they must provide e-learning consumers with high service quality (Pham et al., 2022). Moreover, perceived service quality has been proved to have a significate relationship with satisfaction and continuance intention (Gao et al., 2015; Achmadi and Siregar, 2021; Akdim et al., 2022; Twum et al., 2022).

For e-learning Python courses, service quality is judged to be equally important, as it is a high-level, general-purpose programming language that operates on professional Python programs (Srinath, 2017). In general, an organization always designs an operating window that helps consumers learn and practice. If the service quality is not good, the course will not run smoothly. Thus, the service quality may directly influence whether a Python course can be conducted. The idea is also supported by Stevens et al. (2020) that an ongoing need for quality development is an important challenge in the delivery of e-learning.

Most of the current research on e-learning prefers to focus on university students (Almahamid and Rub, 2011; Pham et al., 2018, 2019; Bae and Shin, 2020; Dwidienawati et al., 2020) and states that there is a significant relationship between service quality and satisfaction. For example, Pham et al. (2018) stated that e-learning service quality positively affects undergraduates' e-learning satisfaction at public universities in the northern US. Further, Dwidienawati et al. (2020) indicated that service quality had a significantly positive impact on university students' e-learning satisfaction at four campuses in Greater Jakarta during the COVID-19 pandemic. Martínez-Argüelles and Batalla-Busquets (2016) explained that perceived service quality has a significant impact on student satisfaction in an online university of Universitat Oberta de Catalunya. Moreover, Lee (2010) proved that the perception of online support service quality significantly affected online learning satisfaction for both Korean and American students. However, Gao et al. (2015) proved that service quality did not affect Chinese consumers' satisfaction with mobile purchases. The relationship between service quality and satisfaction must be examined again, as this study focuses on the different contexts of online Python courses. Therefore, based on the above, Hypothesis 2 is proposed.

**H2**. Perceived service quality positively affects satisfaction.

### Perceived Convenience and Satisfaction

Convenience represents the idea that individuals estimate the time and effort required to achieve a goal whenever deciding upon something. Consumers naturally seek and value products and services that are conveniently available and easy to use to reduce the psychological/physical costs and the physical/mental efforts involved with decision making (Sanford et al., 2017). In e-learning, Sanford et al. (2017) explained that convenience is the flexibility and ease with which a student is allowed to participate in an online class. Truong et al. (2017) pointed out that the convenience of e-learning is related to saving time and energy, including physical and mental work. To sum up, the convenience of e-learning means that online courses have great freedom and flexibility, which can help consumers save time and energy.

Perceived convenience has become the hottest topic widely discussed in e-learning over the last few years (Truong et al., 2017; Bansah and Agyei, 2022; Sugandini and Istanto, 2022). It can be one of the influential factors contributing to the continuance intention (Chang et al., 2013). Current studies have discussed the relationship between perceived convenience and satisfaction, although the relationship is found to be inconsistent. For example, Sanford et al. (2017) stated that students strongly associated convenience with online course satisfaction in AACSB-accredited undergraduate business programs in the eastern US. They also found that convenience may be a factor that generally motivates students across all course formats. On the contrary, Ibrahim and Hidayat-Ur-Rehman (2021) explained that the convenience of virtual classes negatively affected undergraduate and postgraduate students' satisfaction in Saudi Arabia, Egypt, and Pakistan during COVID-19. Thus, in different contexts, the relationship might change.

This research focuses on e-learning Python courses in China, which warrants a re-examination of the relationship between perceived convenience and satisfaction. Hence, based on the above, Hypothesis 3 is proposed.

**H3**. Perceived convenience positively affects satisfaction.

### Perceived Usefulness and Satisfaction

Perceived usefulness is defined as the degree to which a person believes that using a particular system will increase their job performance (Daneji et al., 2019; Natasia et al., 2022). It is one of the potential factors in the process of consumers' continuance intention (Loh et al., 2022; Saima et al., 2022). Consumers' intention to use it for learning in the future depends on their perception of its usefulness (Bouyzem et al., 2022).

The existing research also finds a relationship between perceived usefulness and satisfaction. Some research stated that increased perceived usefulness could increase satisfaction and success in using e-learning (Sugandini and Istanto, 2022). For example, Ibrahim and Hidayat-Ur-Rehman (2021) stated that the perceived usefulness of virtual classes positively affected undergraduate and postgraduate students' satisfaction in Saudi Arabia, Egypt, and Pakistan during COVID-19. Almahamid and Rub (2011) proposed that a high level of perceived usefulness has to be maintained to increase students' satisfaction with the e-learning system in Jordan. However, some research pointed out that there is no relationship between perceived usefulness and satisfaction. For instance, Daneji et al. (2019) argued that perceived usefulness has no significant effect on undergraduate students' satisfaction with massive open online courses in Malaysian public universities. Thus, a change in context might influence the relationship between perceived usefulness and satisfaction.

This research is based on Chinese consumers and will assume a positive relationship between perceived usefulness and satisfaction. The ISC model postulated perceived usefulness as a strong and direct determinant of consumer satisfaction (Bhattacherjee, 2001). Therefore, based on the above, Hypothesis 4 is proposed.

**H4**. Perceived usefulness positively affects satisfaction.

### Satisfaction and Continuance Purchase Intention

Continuance intention is the ultimate goal of the ISC model. It is defined as the intention to continue using an information system (Bhattacherjee, 2001). In marketing, consumers' continuance intention may determine an organization's success (Sasongko et al., 2022). Analyzing consumers' continuance intention in e-learning may help e-learning organizations maintain long-term development. This study focuses on identifying consumers' continuance purchase intention, i.e., consumers' intention to continue paying the fee for the remainder of the Python course. The result of this study may also provide a reference for the e-learning organization.

When it comes to the relationship between satisfaction and continuance intention, current studies explained that satisfaction is more predictive in explaining continuance intention or considered to be one main driver of continuance intention (Akdim et al., 2022; Gunawan et al., 2022; Saima et al., 2022). It is an effective outcome that influences learners' decisions to remain or leave a course (Dal Santo et al., 2022). Ibrahim and Hidayat-Ur-Rehman (2021) supported this finding that undergraduate and postgraduate students' satisfaction positively affected their continuance intention in Saudi Arabia, Egypt, and Pakistan during COVID-19. In addition, Cheng (2020) proved that Taiwanese students' satisfaction with cloud-based e-learning systems within their educational institution would increase their continuance intention. However, there is a lack of empirical research focusing on the relationship between satisfaction and continuance purchase intention in e-learning, as they focus more on continuance intention—this study mainly examines the process of consumers' continuance purchase intention based on the ISC model. Therefore, based on the above, Hypothesis 5 is proposed.

**H5**. Satisfaction positively affects the continuance of purchase intention.

### Self-Efficacy and Continuance Purchase Intention

Self-efficacy can be defined as the confidence in one's ability to perform certain learning tasks in the long term of the e-learning process or to complete tasks in the right time frame (Li et al., 2012; Haq et al., 2022; Muliati et al., 2022). It has been more concerned with e-learning and continuance intention (Chang et al., 2020; She et al., 2021a,b, 2022; Calaguas and Consunji, 2022; Sulaymani et al., 2022).

The relationship between self-efficacy and continuance intention has been widely discussed in the existing literature. However, their relationship has been found to be inconsistent. Some research stated that there is a direct relationship between self-efficacy and continuance intention. For example, Saima et al. (2022) proved that the impact of self-efficacy on mobile financial services users' continuance intention was significant during the COVID-19 pandemic in Bangladesh. Chang et al. (2020) proved that e-learning self-efficacy positively affected adults' continuance intention to use an e-learning website. At the same time, some research indicates that there is an indirect relationship between self-efficacy and continuance intention. For instance, Nguyen and Ha (2022) indicated that self-efficacy indirectly affects Grab users' continuance intention through behavioral adaptation in the platform-based context. In short, the relationship between self-efficacy and continuance intention differs in different contexts.

However, there is a limitation to the empirical research discussing the relationship between self-efficacy and continuance purchase intention in the context of an online Python course. This study focuses on the consumers' continuance purchase intention of the e-learning Python course. One of the aims is to identify the factors influencing the continuance of purchase intention. Thus, the relationship between self-efficacy and continuance purchase intention must be investigated again. Therefore, based on the above, Hypothesis 6 is proposed.

**H6**. Self-efficacy positively affects the continuance of purchase intention.

### E-Word of Mouth and Continuance Purchase Intention

Electronic word of mouth (e-WOM) refers to the information about products, services, or companies shared online in the form of consumers' knowledge, experiences, and opinions that can be either positive or negative (Chen et al., 2012). E-WOM occurs on various online channels such as BBSs, online forums, and virtual communities (Tsimonis and Dimitriadis, 2014). As interaction becomes more prevalent on the Web, more and more consumers make their purchasing decisions based on e-WOM (Sulthana and Vasantha, 2019). Negative e-WOM generally carries more impact than positive e-WOM, making it more significant for organizations globally (Raymond, 2015). As a result, many companies have started to pay attention to and re-examine their corporate policies from a consumer perspective to improve their strategic competition (Chen et al., 2012). Therefore, e-WOM has gradually attracted the attention of the institutions that develop online courses (Shehzadi et al., 2020; Liao et al., 2022).

Most previous research focuses on the relationship between e-WOM continuance intention (Chen et al., 2012; Mentoh and Suki, 2017). However, the existing research explained inconsistent results about the relationship between e-WOM and continuance intention. Some research stated that e-WOM indirectly influences continuance intention. For example, Ma et al. (2019) explained that users' satisfaction mediated e-WOM, influencing continuance intention toward cross-border shopping websites in Taiwan. Moreover, some research indicated a direct relationship between e-WOM and continuance intention. Chen et al. (2012) proved that e-WOM positively affects consumers' continuance intention for the usage of Web 2.0. Simply, the relationship between e-WOM and continuance intention in different contexts.

This research focuses on consumers' continuance purchase intention of e-learning Python courses. The relationship between e-WOM and continuance purchase intention will be tested based on the ISC model. Therefore, based on the above, Hypothesis 7 is proposed.

**H7**. E-word of mouth positively affects the continuance of purchase intention.

### The Mediating Role of Satisfaction

Previous research has shown that satisfaction plays an essential role in determining consumers' continuance purchase intention. For example, Cheng (2020) proved that the course quality positively contributed to their satisfaction with the cloud-based e-learning system, which indirectly led to their continuance intention with the system. In the same vein, Joo and Choi (2016) demonstrated that satisfaction had a mediating effect on the relationship between resource quality and students' continuance intention to use online library resources (OLRs) in the context of academic libraries. Further, users' satisfaction has been found to be a mediator between perceived convenience and perceived usefulness with continuance intentions (Joo and Choi, 2016; Omotayo and Omotope, 2018). Based on the ISC model and previous studies, the current study proposes that consumers' satisfaction with online Python courses may explain the effects of experience courses (including perceived course quality, perceived service quality, perceived convenience, and perceived usefulness) on continuance purchase intention. Therefore, based on the above, the following hypotheses are proposed:

**H8**. Satisfaction mediates the relationship between perceived course quality and the continuance purchase intention.
**H9**. Satisfaction mediates the relationship between perceived service quality and the continuance purchase intention.
**H10**. Satisfaction mediates the relationship between perceived convenience and the continuance purchase intention.
**H11**. Satisfaction mediates the relationship between perceived usefulness and the continuance purchase intention.

## Methods

### Research Design and Participant

This study used a correlational cross-sectional, questionnaire-based research design to examine the factors that affect the consumers' satisfaction and continuance purchase intention of the e-learning Python course among Chinese consumers. An online survey was conducted and distributed to collect data from Chinese consumers who enrolled in paid online Python courses using the online survey web SoJump. The online survey link, with a short description of the objective of the study, was shared through the social media app—WeChat. The inclusion criteria for participation in this study were as follows: (1) Chinese consumers who had enrolled in paid online Python courses (screened using a self-reported question as “indicate whether you had the experience of taking paid online Python courses”), and (2) those who willingly participated in this study. In total, using the convenient sampling technique, 796 consumers have participated in the survey, and 508 respondents have completed the survey and fulfilled the inclusion criteria of this study. The sample of this study consisted of 240 males (47.2%), and 268 females (52.8%). Most of the participants were aged 45 years and below (74.1%), and the majority of them (90.6%) had at least an undergraduate degree. The details of the respondents' demographic profiles are shown in Table 1.

**Table 1:** Demographic profiles of the respondents.

**Characteristics**		**Frequency (n)**	**Percentage (%)**
**Gender**	Male	240	47.2
	Female	268	52.8
**Age**	25 and below	100	19.7
	26–35	172	33.9
	36–45	104	20.5
	46–55	83	16.3
	56 and above	49	9.6
**Monthly income**	Under 2,500 yuan	168	33.6
	2,501–3,500 yuan	48	9.6
	3,501–4,500 yuan	90	18
	4,501–5,500 yuan	34	6.8
	5,501–6,500 yuan	120	24
	6,501 yuan and above	12	2.4
**Occupational status**	Private sector	250	49.2
	Public sector	110	21.7
	Retired	34	6.7
	Unemployed	30	5.9
	Students	84	16.5
**Education level**	Secondary school and lower	36	7.1
	Diploma	183	36
	Undergraduate	232	45.7
	Postgraduate	57	11.2

### Measures

#### Perceived Course Quality

To measure consumers' perceived course quality of paid online Python courses, we adapted and modified a 3-item scale by Li et al. (2012) to fit online Python learning. Respondents were asked to indicate their level of agreement with each of three statements about the online Python course (e.g., “the content of the online Python course provides abundant information and problem-solving techniques.”). Each item was recorded on a 7-point Likert scale ranging from 1 (strongly disagree) to 7 (strongly agree).

#### Perceived Service Quality

To measure consumers' perceived service quality toward paid online Python courses, we adapted a 4-item scale from Li et al. (2012). On a 7-point scale ranging from 1 (strongly disagree) to 7 (strongly agree), respondents were requested to indicate agreement with four statements (e.g., “The Python online learning operating system has enhanced learning efficiency”).

#### Perceived Convenience

This study adapted a 4-item scale developed by Yoon and Kim (2007) to measure consumers' perceived convenience of paid online Python courses. The scale was modified to be used in the current context (e.g., wireless LAN was replaced by the online Python course). Respondents were asked to indicate their level of agreement with each of four statements (e.g., “I find online Python courses convenient for my learning”). Each item was recorded on a 7-point Likert scale ranging from 1 (strongly disagree) to 7 (strongly agree).

#### Perceived Usefulness

This study measured consumers' perceived usefulness of paid online Python courses using three items from Alsabawy et al. (2016). Respondents were asked how much they agree that using the knowledge learnt from online Python increases their productivity, improves their performance, and enables them to accomplish their tasks more quickly. The response was scored on a 7-point Likert scale, ranging from 1 (strongly disagree) to 7 (strongly agree).

#### Satisfaction

A 3-item scale was adapted to measure consumers' satisfaction with paid online Python courses (Chiu et al., 2005). Respondents were asked how much they were satisfied with the three statements regarding the paid online Python course (e.g., I am satisfied with the performance of the online Python course), with responses recorded on a 7-point Likert scale ranging from 1 (strongly disagree) to 7 (strongly agree).

#### Self-Efficacy

Following Pavlou and Fygenson (2006), this study used two statements to measure consumers' self-efficacy regarding their confidence in the study paid online Python course (e.g., “If I want to, I am confident I could finish the online Python course on time”) on a 7-point Likert scale ranging from 1 (strongly disagree) to 7 (strongly agree).

#### E-Word of Mouth (e-WOM)

The e-WOM was measured by a 3-item from Park and Lee (2009) on a 7-point Likert scale of agreement, from 1 (strongly disagree) to 7 (strongly agree), with the statements regarding the paid online Python course (e.g., “I will refer to e-WOM information in a purchase decision on a paid online Python course”).

#### Continuance Purchase Intention

To measure consumers' continuance purchase intention on paid online Python courses, a 3-item scale was adapted from Chiu et al. (2005). Respondents were asked to indicate whether they agreed with each of three statements (e.g., “I intend to continue to purchase the online Python course in the future”). Each statement was recorded on a 7-point Likert scale ranging from 1 (strongly disagree) to 7 (strongly agree).

### Data Analysis

This study used variance-based Partial Least Squares Structural Equation Modeling (PLS-SEM), and SmartPLS software version 3.3.2 was used to perform the data analysis. PLS-SEM does not require any distribution assumptions and maximizes the explained variance of the proposed model (Sharif and Nia, 2018; She et al., 2021a,b, 2022). Moreover, PLS-SEM allows users to evaluate more complex models with numerous variables, indicator constructs, and structural paths (She et al., 2022). Additionally, SmartPLS software offers a wide range of algorithmic and modeling possibilities and professional support (Ringle et al., 2015). Following the two-step approach, this study first assessed the measurement model and then the structural model. To assess the measurement model, the confirmatory factor analysis (CFA) was conducted to evaluate model fit, internal consistency, and construct reliability and validity. The model fit was assessed using a standardized root mean squared residual (SRMR) of >0.08 (Pavlov et al., 2020). Items with a factor loading of <0.5 were excluded. Cronbach's alpha and composite reliability (CR) of >0.7 were used to evaluate internal consistency and construct reliability (Hair et al., 2014). Construct validity was assessed through convergent and discriminant validity. To establish convergent validity, the CR should be >0.7, and the average variance extracted (AVE) should be >0.5 and less than its respective CR (Fornell and Larcker, 1981; She et al., 2021b). In terms of discriminant validity, this study followed the Fornell–Larcker criterion, where the square root of the AVE for each construct should be greater than its correlation with other constructs (Fornell and Larcker, 1981). In the second step, the proposed structural model and hypothesis were assessed using a bias-corrected bootstrapping technique with 5,000 replications. All tests in this study were two-tailed, and a *p*-value of <0.05 was considered to be statistically significant.

## Results

Initially, this study assessed the measurement model by performing CFA. The results showed that the measurement model fits the data well, as evidenced by SRMR (0.037) being >0.08. The results of the measurement model assessment are shown in Table 2. The factor loadings for all items were >0.7 and statistically significant. For all constructs, Cronbach's alpha (ranging from 0.791 to 0.896) and CR (ranging from 0.896 to 0.928) were >0.7, indicating good internal consistency and construct reliability. Also, the AVEs for all constructs were >0.5 (ranging from 0.742 to 0.827), providing support for convergent validity. In terms of discriminant validity, results showed that the square root of each construct's AVE was greater than its correlation with other constructs (Table 3), indicating good discriminant validity.

**Table 2:** Measurement model assessment.

**Constructs**	**Items**	**Outer loadings**	**Cronbach's alpha**	**Composite reliability**	**AVE**
**Perceived course quality (PCQ)**			0.826	0.896	0.742
	The content of the online Python course provides abundant information and problem-solving techniques.	0.851			
	The online Python course content is interesting and practical.	0.875			
	The methods of evaluation and assessment of the online Python course are appropriate.	0.858			
**Perceived service quality (PSQ)**			0.889	0.923	0.749
	The operating system of the online Python course has enhanced the learning efficiency.	0.858			
	I believe the staff in charge of the online Python course are aware of my concerns.	0.872			
	I find the operating system of the online Python course is easy for me to check my learning problem.	0.859			
	Tutors of the online Python course show a sincere interest in solving my problems.	0.874			
**Perceived convenience (PC)**			0.896	0.928	0.763
	Using the online Python course system enables me to accomplish my study at a convenient time.	0.876			
	I can perform my learning anyplace with the use of the online Python course system.	0.865			
	Using an online Python course system gives me convenience in performing my learning.	0.878			
	I find the online Python course system convenient for my learning.	0.874			
**Perceived usefulness (PU)**			0.853	0.910	0.772
	Using the knowledge from the online Python course increases my productivity.	0.883			
	Using the knowledge from the online Python course improves my job performance	0.878			
	Using the knowledge from the online Python course enables me to accomplish my tasks more quickly	0.875			
**Satisfaction**			0.862	0.916	0.784
	I am satisfied with the performance of the online Python course.	0.884			
	I am pleased with the experience of using the online Python course.	0.889			
	My decision to use the online Python course service was a wise one	0.884			
**Self-efficacy (SE)**			0.791	0.905	0.827
	If I wanted to, I would be able to insist on the online Python course every day.	0.908			
	If I want to, I am confident I can finish the online Python course on time.	0.911			
**E-WOM**			0.848	0.908	0.767
	I will refer to e-WOM information in a purchase decision for an online Python course.	0.877			
	Overall, I think e-WOM information is credible.	0.883			
	This e-WOM information will crucially affect my purchase decision on an online Python course.	0.868			
**Continuance purchase intention (CPI)**			0.856	0.912	0.776
	I intend to continue purchasing the online Python course in the future.	0.880			
	I will continue to purchase the online Python course in the future.	0.887			
	I will continue to purchase the remainder of the online Python course in the future.	0.876			

**Table 3:** Discriminant validity assessment using Fornell–Larcher criterion.

**Construct**	**(1)**	**(2)**	**(3)**	**(4)**	**(5)**	**(6)**	**(7)**	**(8)**
PCQ	0.861							
PSQ	0.839	0.866						
PC	0.844	0.852	0.873					
PU	0.829	0.835	0.861	0.879				
Satisfaction	0.818	0.853	0.843	0.852	0.885			
SE	0.804	0.804	0.814	0.788	0.772	0.909		
E-WOM	0.839	0.864	0.857	0.836	0.846	0.817	0.876	
CPI	0.829	0.852	0.867	0.848	0.848	0.797	0.840	0.881

**Figure 2:**
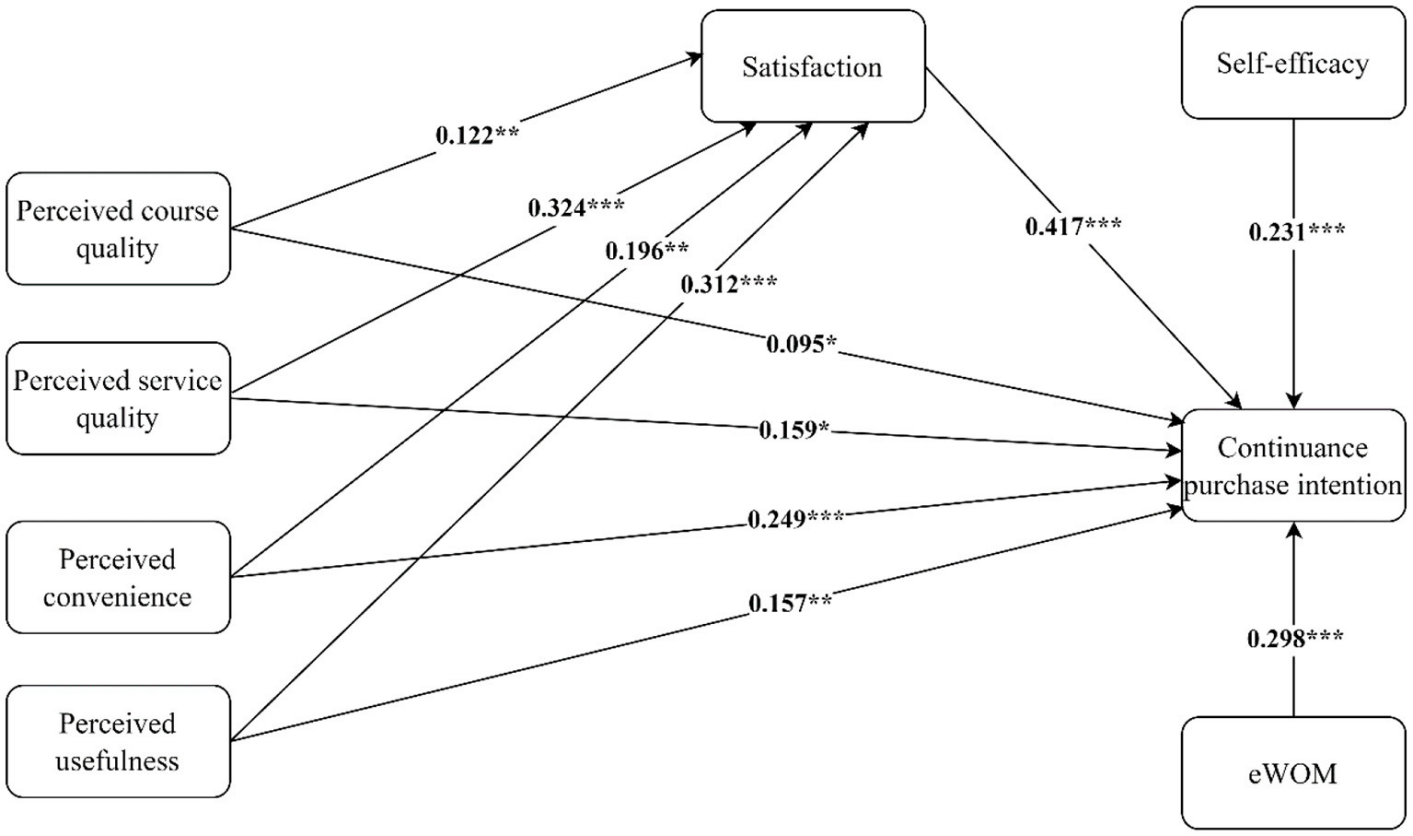
The results of the structural model. ***p < 0.001, **p < 0.01, *p < 0.05.

Next, the proposed model and hypotheses were tested when controlling for the effect of participants' age, gender, position, income, and education level. Table 4 reports the results of the structural model assessment and hypotheses testing. The results of testing the direct effects showed a significant positive effect of perceived course quality on satisfaction (β = 0.122, *t* = 2.829, *p* < 0.01), perceived service quality on satisfaction (β = 0.324, *t* = 5.233, *p* < 0.001), perceived convenience on satisfaction (β = 0.196, *t* = 3.231, *p* < 0.01), perceived usefulness on satisfaction (β = 0.312, *t* = 6.085, *p* < 0.001), as well as a significant positive effect of satisfaction on continuance purchase intention (β = 0.417, *t* = 7.038, *p* < 0.001), which supported H1, H2, H3, H4, and H5. The results also showed the significnat positive effect of self-efficay on continuance purchase intention (β = 0.231, *t*-value = 5.215, *p* < 0.001), and significnat positive effect of e-WOM on continuance purchase intention (β = 0.298, *t*-value = 5.702, *p* < 0.001), providing support for H6 and H7. Moreover, the results from testing the indirect effects showed that perceived course quality (β = 0.022, *t*-value = 2.167, *p* < 0.05), perceived service quality (β = 0.057, *t*-value = 3.172, *p* < 0.01), perceived convenience (β = 0.034, *t*-value = 2.095, *p* < 0.05), and perceived usefulness (β = 0.055, *t*-value = 3.059, *p* < 0.01) indirectly affects continuance purchase intention through satisfaction, providing support for H8, H9, H10, and H11. The significant positive relationship between perceived course quality and continuance purchase intention (β = 0.095, *t* = 2.052, *p* < 0.05), perceived service quality and continuance purchase intention (β = 0.159, *t* = 2.500, *p* < 0.05), perceived convenience and continuance purchase intention (β = 0.249, *t* = 4.071 *p* < 0.01), perceived usefulness and continuance purchase intention (β = 0.157, *t* = 2.864, *p* < 0.01) in the mediation model, indicating that all the mediation effects were partial. The model explained 80.7% of the total variance of satisfaction and 78.9% of the total variance of continuance purchase intention. Figure 2 depicts the results of the path coefficient for all paths.

**Table 4:** The results of the structural model assessment and hypotheses testing.

**Paths**	**Path coefficients** **(*t*-value)**	**95% confidence intervals**	**Hypotheses**	**Decision**
Direct effect				
Perceived course quality → Satisfaction	0.122^**^ (2.829)	(0.036, 0.205)	H1	Supported
Perceived service quality → Satisfaction	0.324^***^ (5.233)	(0.202, 0.449)	H2	Supported
Perceived convenience → Satisfaction	0.196^**^ (3.231)	(0.073, 0.309)	H3	Supported
Perceived usefulness → Satisfaction	0.312^***^ (6.085)	(0.207, 0.415)	H4	Supported
Satisfaction → Continuance purchase intention	0.417^***^ (7.038)	(0.300, 0.529)	H5	Supported
Self-efficacy → Continuance purchase intention	0.231^***^ (5.215)	(0.141, 0.318)	H6	Supported
E-WOM → Continuance purchase intention	0.298^***^ (5.702)	(0.203, 0.409)	H7	Supported
Mediation effect				
Perceived course quality → Satisfaction → Continuance purchase intention	0.022^*^ (2.167)	(0.005, 0.044)	H8	Supported
Perceived service quality → Satisfaction → Continuance purchase intention	0.057^**^ (3.172)	(0.023, 0.094)	H9	Supported
Perceived convenience → Satisfaction → Continuance purchase intention	0.034^*^ (2.095)	(0.007, 0.070)	H10	Supported
Perceived usefulness → Satisfaction → Continuance purchase intention	0.055^**^ (3.059)	(0.021, 0.091)	H11	Supported

*** *p < 0.001;*

** *p < 0.01;*

* *p < 0.05*.

## Discussion

### Findings

This study helped understand the process of consumers' continuance purchase intention in the e-learning industry. It identified the factors that influence consumers' satisfaction and continuance purchase intention of e-learning Python courses. The results found that perceived course quality has a positive relationship with consumers' satisfaction, suggesting a greater satisfaction with the higher perceived course quality of e-learning Python courses. This finding is consistent with previous research that course quality significantly contributes to consumer satisfaction (Li et al., 2012; Cheng, 2020).

Perceived service quality positively influences consumers' satisfaction with the e-learning Python courses. This means that the higher the perceived service quality of e-learning Python courses, the more satisfied the consumers will be. This is partially similar to Pham et al. (2018) and Dwidienawati et al. (2020), who indicated that service quality significantly impacts university students' e-learning satisfaction.

This study also found a positive relationship between perceived convenience and consumers' satisfaction. This finding is consistent with the findings of Sanford et al. (2017), who found that students strongly associate convenience with online course satisfaction. They also suggested that convenience may be a factor that generally motivates students across all course formats.

Next, this study found that perceived usefulness positively affects consumers' satisfaction. It means if consumers perceive more usefulness from the Python course, they will be more satisfied with it. This finding is consistent with the ISC model, which explains that perceived usefulness determines consumer satisfaction (Bhattacherjee, 2001).

Also, the finding reveals that satisfaction has a positive effect on consumers' continuance purchase intention of the e-learning of the Python course. This finding is consistent with the mechanism of the ISC model, providing additional evidence to support the theory of ISC.

Moreover, self-efficacy is positively related to consumers' continuance purchase intention of the e-learning of the Python course. It means that self-efficacy would be one important factor that affects consumers' intention to continue the purchase (availing) of the Python course. This result is partially comparable to that of Chang et al. (2020), who demonstrated that self-efficacy positively affected adults' continuance intention to use e-learning websites. The results also demonstrated a significant relationship between e-WOM and continuance purchase intention, which suggests that continuance purchase intention of e-learning Python courses would be affected by the information available online by other users/consumers. This is consistent with the findings of previous research that proved that more and more consumers would make their purchasing decisions based on e-WOM (Sulthana and Vasantha, 2019).

Finally, the findings of this study also confirmed the mediating role of satisfaction in the effects of perceived course quality, perceived service quality, perceived convenience, and perceived usefulness on consumers' continuance purchase intention of the online Python course in China. The findings explain how perceived course quality, perceived service quality, perceived convenience, perceived usefulness, and continuance purchase intention are related to satisfaction, suggesting that satisfaction explains how these variables are associated. The results support the ISC model, indicating satisfaction played a mediating role in explaining continuance intention (Bhattacherjee, 2001). The mediation effect demonstrated the critical importance of satisfaction in influencing the consumers' continuance purchase intention. This indicates that consumers' perceived course quality, perceived service quality, perceived convenience, and perceived usefulness of the experience course will significantly influence their satisfaction, affecting their continuance purchase intention for the remainder of the Python course.

In short, this study found that perceived course quality, service quality, convenience, and usefulness are important factors that affect consumers' satisfaction with e-learning Python courses. It also found that satisfaction, self-efficacy, and e-WOM can predict consumers' continuance purchase intention for the remainder of their courses. Moreover, it found that satisfaction mediates the relationship between perceived course quality, service quality, convenience, usefulness, and continuance purchase intention, which in this context is the e-learning Python course.

### Theoretical Implications

One crucial theoretical contribution is that this study extends the current ISC model into a new area of consumers' continuance purchase intention in e-learning. This study has identified more factors that affect satisfaction and continuance intention based on the ISC model.

Although past studies have revealed the factors influencing consumers' satisfaction and continuance intention, consumers' purchase intention has not been widely studied. It contributes to the success of the course or users' satisfaction with the organization (Peña-García et al., 2020). Therefore, understanding the predictors or factors that influence the consumers' satisfaction and continuance intention is important because it enhances how to stimulate consumers' continuance purchase intention.

This study also contributes to the existing literature on continuance intention. To the authors' best knowledge, this study may be the first to identify the process of consumers' continuance purchase intention and have an intensive analysis of the two different stages (experience course and the remainder of the course) of an e-learning course. It supports the ISC model to explain the process of continuance purchase intention.

This study tested the mediating role of satisfaction on consumers' perceived course quality, perceived service quality, perceived convenience, perceived usefulness, and continuance purchase intention, specifically in the context of the online Python courses. The findings of this study contribute to the body of knowledge in this field and shed new insight on the relationship between consumers' perception and continuance purchase intention by indicating that these relationships are primarily mediated by satisfaction. By applying this mediation model, we improve our understanding of the significant role of satisfaction in such relationships in this research among Chinese e-learning consumers.

### Practical Implication of Research

This study provides several implications for the e-learning organization from a practical perspective. First, this research model explained the process of consumers' continuance purchase intention. Most e-learning organizations divide their courses into two parts: the experience course and the remainder of the course. In doing so, they expect to attract more consumers' attention through the experience course and aim to satisfy them so that, in turn, it changes their intention to continuance purchase in their favor. Thus, this research will help e-learning organizations have a deep understanding of consumers' purchase behavior and may provide useful information for e-learning organizations to predict consumers' continuance purchase intention.

Besides, perceived course quality, service quality, convenience, and usefulness have been found to be important factors that influence consumers' satisfaction with e-learning. Organizations may focus on these aspects to make consumers more satisfied. For instance, designing easy-to-understand courses for beginners and setting flexible learning hours and appropriate timings of the day may give consumers more convenience.

Moreover, this research found that satisfaction, self-efficacy, and e-WOM have a crucial role to play in determining a consumer's continuance purchase intention for the remainder of the course, which in this context is the e-learning Python course. Therefore, organizations can invest more in increasing consumers' satisfaction with e-learning Python courses, affecting consumers' continuance purchase intention. Also, the organization can facilitate the consumers' continuance purchase intention by increasing individual self-efficacy. Lastly, organizations should exercise particular care with the external views that may influence consumers' decision of continuance purchase.

Finally, satisfaction has been found to play an important mediation in consumers' continuance purchase intention. The effect of perceived course quality, service quality, convenience, and usefulness on consumers' continuance purchase intention will be increased by customer satisfaction. Thus, organizations may focus on the consumers' satisfaction with the experience course, influencing consumers' continuance purchase intention for the remainder of the course. By doing this, organizations may want to learn more about consumers' evaluation of the experience course, interact with consumers to improve mutual understanding of consumers and do their best to meet consumers' expectations, thus, building and increasing consumer satisfaction. As satisfaction increased, it would help maximize the effect on consumers' perception of continuance purchase intention and better predict consumers' continuance purchase intention.

### Limitations and Future Research

Although this study has several interesting findings, certain limitations were noted. First, although this study has suggested that perceived course quality, perceived service quality, perceived convenience, and perceived usefulness are important factors affecting consumers' satisfaction with e-learning Python courses, in addition, self-efficacy and e-WOM are vital factors influencing consumers' continuance purchase intention, and there may be other important factors. Future studies should include other factors, such as attitude, into the model. Finally, consumer behaviors are influenced by national cultural characteristics (Shavitt and Cho, 2016). Because this study collected data from China, the findings might not be applicable to other countries/regions. Further studies are required to investigate our model in different cultural contexts.

## Conclusion

This study develops a complex and new model based on the ISC model in the online Python courses. The results pointed out that the perceived course quality, perceived service quality, perceived convenience, and perceived usefulness are the factors that influence consumers' satisfaction in the stage of experience course, which affects the consumers' continuance purchase intention. Besides, the findings show that satisfaction, self-efficacy, and e-word of mouth decide the consumers' continuance purchase intention for the reminder course. Moreover, this study proved that satisfaction is an important factor that mediates the relationship between perceived course quality, perceived service quality, perceived convenience, and perceived usefulness with continuance purchase intention. This study provides a reference for the academy and provides guidelines for the e-learning organization.

## Data Availability Statement

The raw data supporting the conclusions of this article will be made available by the authors, without undue reservation.

## Ethics Statement

The study involving human participants was reviewed and approved by the Ethics Committee of Taylor's University, Malaysia. The participants provided their written informed consent to participate in this study.

## Author Contributions

JZ contributed to the study conception and design. Material preparation and data collection were performed by JZ and DW. LS performed the data analysis and interpreted the results. The first draft of the manuscript was written by JZ, LS, DW, and AS. All authors commented on previous versions of the manuscript. All authors read and approved the final manuscript.

## Conflict of Interest

The authors declare that the research was conducted in the absence of any commercial or financial relationships that could be construed as a potential conflict of interest.

## Publisher's Note

All claims expressed in this article are solely those of the authors and do not necessarily represent those of their affiliated organizations, or those of the publisher, the editors and the reviewers. Any product that may be evaluated in this article, or claim that may be made by its manufacturer, is not guaranteed or endorsed by the publisher.

## Footnotes

^1^Python is an interpreted, high-level, general-purpose programming language. It can be used for handing different packages with a wide range of functionality, including automation, data analytics, databases, documentation graphical user interfaces, image processing, machine learning, mobile app, computer networking, scientific computing, system administration, text processing, etc.

## References

[B1] 51CTO (2021). What Do You Use Python Mainly For? This Demand Ranks First. Available online at: https://blog.51cto.com/u_15091060/2680954 (accessed August 16, 2021).

[B2] Achmadi A. Siregar A. O. (2021). The effect of system quality, information quality and service quality on user satisfaction of e-learning system. Int. J. Bus. Rev. 4, 103–120. 10.17509/tjr.v4i2.4048321543971

[B3] Aisoutu (2022). Summary of programming Education Policies Across the Country. Available online at: https://www.aisoutu.com/a/1510817 (accessed January 26, 2022).

[B4] Akdim K. Casal,ó L. V. Flavián C. (2022). The role of utilitarian and hedonic aspects in the continuance intention to use social mobile apps. J. Retail. Consum. Serv. 66, 102888. 10.1016/j.jretconser.2021.102888

[B5] Almahamid S. Rub F. A. (2011). “Factors that determine continuance intention to use e-learning system: an empirical investigation.” in International Conference on Telecommunication Technology and Applications Proc. of CSIT (Singapore).

[B6] Alsabawy A. Y. Cater-Steel A. Soar J. (2016). Determinants of perceived usefulness of e-learning systems. Comput. Hum. Behav. 64, 843–858. 10.1016/j.chb.2016.07.065

[B7] Bae J. H. Shin H. Y. (2020). A comparative study on e-learning satisfaction between Korea and China. J. Dig. Converg. 18, 369–377. 10.14400/JDC.2020.18.1.369

[B8] Bansah A. K. Agyei D. D. (2022). Perceived convenience, usefulness, effectiveness and user acceptance of information technology: evaluating students' experiences of a Learning Management System. Technol. Pedagogy Educ. [Epub ahead of print]. 10.1080/1475939X.2022.2027267

[B9] Bhattacherjee A. (2001). Understanding information systems continuance: an expectation-confirmation model. MIS Q. 25, 351–370. 10.2307/3250921

[B10] Bøe T. (2018). E-learning technology and higher education: the impact of organizational trust. Tert. Educ. Manag. 24, 362–376. 10.1080/13583883.2018.1465991

[B11] Bouyzem M. Ghilane H. Moustakim O. Tsouli D. (2022). Higher education in Morocco in the Covid-19 era: what perception of the usefulness and ease of use of e-learning? IJBTSR Int. J. Bus. Technol. Stud. Res. 3, 7. 10.5281/zenodo.5849055

[B12] Budner P. Fischer M. Rosenkranz C. Basten D. Terlecki L. (2017). Information system continuance intention in the context of network effects and freemium business models: a replication study of cloud services in Germany. AIS Trans. Replication Res. 3, 4. 10.17705/1atrr.00019

[B13] Cai J. Zhao Y. Sun J. (2022). Factors influencing fitness app users' behavior in China. Int. J. Hum. Comput. Interact. 38, 53–63. 10.1080/10447318.2021.1921483

[B14] Calaguas N. P. Consunji P. M. P. (2022). A structural equation model predicting adults' online learning self-efficacy. Educ. Inf. Technol. [Epub ahead of print]. 10.1007/s10639-021-10871-y PMC8727476 35002467

[B15] Chang C. C. Liang C. Chiu Y. C. (2020). Direct or indirect effects from “perceived characteristic of innovation” to “intention to pay”: mediation of continuance intention to use e-learning. J. Comput. Educ. 7, 511–530. 10.1007/s40692-020-00165-6

[B16] Chang C. C. Tseng K. H. Liang C. Yan C. F. (2013). The influence of perceived convenience and curiosity on continuance intention in mobile English learning for high school students using PDAs. Technol., Educ. 22, 373–386. 10.1080/1475939X.2013.802991

[B17] Chen S. C. Yen D. C. Hwang M. I. (2012). Factors influencing the continuance intention to the usage of Web 2.0: an empirical study. Comput. Hum. Behav. 28, 933–941. 10.1016/j.chb.2011.12.014

[B18] Cheng Y. M. (2020). Students' satisfaction and continuance intention of the cloud-based e-learning system: roles of interactivity and course quality factors. Educ. Train. 62, 1037–1059. 10.1108/ET-10-2019-0245

[B19] Chiu C.-M. Hsu M.-H. Sun S.-Y. Lin T.-C. Sun P.-C. (2005). Usability, quality, value and e-learning continuance decisions. Comput. Educ. 45, 399–416. 10.1016/j.compedu.2004.06.001

[B20] Chow W. S. Shi S. (2014). Investigating students' satisfaction and continuance intention toward e-learning: an extension of the expectation–confirmation model. Proc.-Soc. Behav. Sci. 141, 1145–1149. 10.1016/j.sbspro.2014.05.193

[B21] Coursera (2021). What Is Python Used For? A Beginner's Guide. Available online at: https://www.coursera.org/articles/what-is-Python-used-for-a-beginners-guide-to-using-Python (accessed November 21, 2021).

[B22] Dal Santo L. Peña-Jimenez M. Canzan F. Saiani L. Battistelli A. (2022). The emotional side of the e-learning among nursing students: the role of the affective correlates on e-learning satisfaction. Nurse Educ. Today 110, 105268. 10.1016/j.nedt.2022.105268 35093743

[B23] Daneji A. A. Ayub A. F. M. Khambari M. N. M. (2019). The effects of perceived usefulness, confirmation and satisfaction on continuance intention in using massive open online course (MOOC). Knowl. Manag. E-Learn. 11, 201–214. 10.34105/j.kmel.2019.11.010

[B24] Dwidienawati D. Abdinagoro S. B. Tjahjana D. Gandasari D. (2020). Forced shifting to e-learning during the covid-19 outbreak: information quality, system quality, service quality, and goal orientation influence to e-learning satisfaction and perceived performance. Int. J. Adv. Trends Comput. Sci. Eng. 9, 1518–1525. 10.30534/ijatcse/2020/939220202020

[B25] Erfannia L. Sharifian R. Yazdani A. Sarsarshahi A. Rahati R. Jahangiri S. (2022). Students' satisfaction and e-learning courses in Covid-19 pandemic era: a case study. Stud. Health Technol. Inform. 289, 180–183. 10.3233/SHTI210889 35062122

[B26] Fornell C. Larcker D. F. (1981). Evaluating structural equation models with unobservable variables and measurement error. J. Market. Res. 18, 39–50. 10.1177/002224378101800104

[B27] Franque F. B. Oliveira T. Tam C. de Oliveira Santini F. (2020). A meta-analysis of the quantitative studies in continuance intention to use an information system. Internet Res. 31, 1066–2243. 10.1108/INTR-03-2019-0103

[B28] Gao L. Waechter K. A. Bai X. (2015). Understanding consumers' continuance intention towards mobile purchase: a theoretical framework and empirical study–a case of China. Comput. Hum. Behav. 53, 249–262. 10.1016/j.chb.2015.07.014

[B29] Global Industry Analysts Inc. (2021). MCP-4107: E-Learning—Global Market Trajectory and Analytics Report. Available online at: http://www.strategyr.com/eLEARNING_Online_Education_Market_Report.asp (accessed August 19, 2021).

[B30] Gunawan F. Santoso A. S. Yustina A. I. Rahmiati F. (2022). Examining the effect of radical innovation and incremental innovation on leading e-commerce startups by using expectation confirmation model. Proc. Comput. Sci. 197, 393–402. 10.1016/j.procs.2021.12.155

[B31] Hair J. F. Black W. C. Babin B. J. Anderson R. E. (2014). Multivariat Data Analysis, 7th Edn. Essex: Pearson Education Limited.

[B32] Haq S. Asbari M. Novitasari D. Abadiyah S. (2022). The homeschooling head performance: how the role of transformational leadership, motivation, and self-efficacy? Int. J. Soc. Manag. Stud. 3, 167–179. 10.5555/ijosmas.v3i1.96

[B33] Haythornthwaite C. (2015). Rethinking learning spaces: networks, structures, and possibilities for learning in the twenty-first century. Commun. Res. Pract. 1, 292–306. 10.1080/22041451.2015.1105773

[B34] Hong J. C. Tai K. H. Hwang M. Y. Kuo Y. C. Chen J. S. (2017). Internet cognitive failure relevant to users' satisfaction with content and interface design to reflect continuance intention to use a government e-learning system. Comput. Hum. Behav. 66, 353–362. 10.1016/j.chb.2016.08.044

[B35] Hsu C. L. Yu C. C. Wu C. C. (2014). Exploring the continuance intention of social networking websites: an empirical research. Inf. Syst. e-Bus. Manag. 12, 139–163. 10.1007/s10257-013-0214-3

[B36] Ibrahim Y. Hidayat-Ur-Rehman I. (2021). COVID-19 crisis and the continuous use of virtual classes. Int. J. Adv. Appl. Sci. 8, 117–129. 10.21833/ijaas.2021.04.01432533676

[B37] Jiemian (2017). Python, Which Unifies the AI Industry, is Going to Enter the Elementary School Classroom. Available online at: https://www.jiemian.com/article/1812163.html (accessed September 20, 2021).

[B38] Joo S. Choi N. (2016). Understanding users' continuance intention to use online library resources based on an extended expectation-confirmation model. Electron. Libr. 34, 554–571. 10.1108/EL-02-2015-0033

[B39] Kimiloglu H. Ozturan M. Kutlu B. (2017). Perceptions about and attitude toward the usage of e-learning in corporate training. Comput. Hum. Behav. 72, 339–349. 10.1016/j.chb.2017.02.062

[B40] Lee J. W. (2010). Online support service quality, online learning acceptance, and student satisfaction. Internet High. Educ. 13, 277–283. 10.1016/j.iheduc.2010.08.002

[B41] Li Y. Duan Y. Fu Z. Alford P. (2012). An empirical study on behavioural intention to reuse e-learning systems in rural China. Br. J. Educ. Technol. 43, 933–948. 10.1111/j.1467-8535.2011.01261.x

[B42] Liao Y. K. Wu W. Y. Le T. Q. Phung T. T. T. (2022). The integration of the technology acceptance model and value-based adoption model to study the adoption of e-learning: the moderating role of e-WOM. Sustainability 14, 815. 10.3390/su14020815

[B43] Liu I. F. Chen M. C. Sun Y. S. Wible D. Kuo C. H. (2010). Extending the TAM model to explore the factors that affect intention to use an online learning community. Comput. Educ. 54, 600–610. 10.1016/j.compedu.2009.09.009

[B44] Liu Y. Xie X. Lv J. Jie X. (2020). “Tour guide online independent learning study from the virtual community perspective.” in 2020 International Conference on E-Commerce and Internet Technology (ECIT) (Zhangjiajie, China). 10.1109/ECIT50008.2020.00071

[B45] Loh X. M. Lee V. H. Hew T. S. Lin B. (2022). The cognitive-affective nexus on mobile payment continuance intention during the COVID-19 pandemic. Int. J. Bank Market. [Epub ahead of print]. 10.1108/IJBM-06-2021-0257

[B46] Ma Y. Ruangkanjanases A. Chen S. C. (2019). Investigating the impact of critical factors on continuance intention towards cross-border shopping websites. Sustainability 11, 5914. 10.3390/su11215914

[B47] Martínez-Argüelles M. J. Batalla-Busquets J. M. (2016). Perceived service quality and student loyalty in an online university. Int. Rev. Res. Open Dis. Learn. 17, 264–279. 10.19173/irrodl.v17i4.2518

[B48] Mentoh M. A. B. Suki N. M. (2017). Conceptual study of the impacts of electronic-words-of-mouth (E-WOM) on consumers' continuance intention and brand loyalty of Islamic insurance (Takaful). Adv. Sci. Lett. 23, 8446–8449. 10.1166/asl.2017.9908

[B49] Muliati L. Asbari M. Nadeak M. Novitasari D. Purwanto A. (2022). Elementary school teachers performance: how the role of transformational leadership, competency, and self-efficacy? Int. J. Soc. Manag. Stud. 3, 158–166. 10.5555/ijosmas.v3i1.97

[B50] Natasia S. R. Wiranti Y. T. Parastika A. (2022). Acceptance analysis of NUADU as e-learning platform using the Technology Acceptance Model (TAM) approach. Proc. Comput. Sci. 197, 512–520. 10.1016/j.procs.2021.12.168

[B51] Nguyen D. G. Ha M. T. (2022). What makes users continue to want to use the digital platform? Evidence from the ride-hailing service platform in Vietnam. SAGE Open 12, 21582440211069146. 10.1177/21582440211069146

[B52] Nugroho M. A. Setyorini D. Novitasari B. T. (2019). The role of satisfaction on perceived value and e-learning usage continuity relationship. Proc. Comput. Sci. 161, 82–89. 10.1016/j.procs.2019.11.102

[B53] Omotayo F. O. Omotope A. R. (2018). Determinants of continuance intention to use online shops in Nigeria. J. Internet Bank. Commer. 23, 1–48.

[B54] Park C. Lee T. M. (2009). Information direction, website reputation and e-WOM effect: a moderating role of product type. J. Bus. Res. 62, 61–67. 10.1016/j.jbusres.2007.11.017

[B55] Pavlou P. A. Fygenson M. (2006). Understanding and predicting electronic commerce adoption: an extension of the theory of planned behavior. MIS Q. 30, 115–143. 10.2307/25148720

[B56] Pavlov G. Maydeu-Olivares A. Shi D. (2020). Using the standardized root mean squared residual (SRMR) to assess exact fit in structural equation models. Educ. Psychol. Meas. 81, 110–130. 10.1177/0013164420926231 33456064 PMC7797960

[B57] Peña-García N. Gil-Saura I. Rodríguez-Orejuela A. Siqueira-Junior J. R. (2020). Purchase intention and purchase behavior online: a cross-cultural approach. Heliyon 6, e04284. 10.1016/j.heliyon.2020.e04284 32613132 PMC7322128

[B58] Pham L. Kim K. Walker B. DeNardin T. Le H. (2022). “Development and validation of an instrument to measure student perceived e-learning service quality.” in Research Anthology on Service Learning and Community Engagement Teaching Practices, ed Information Resources Management Association (Hershey, PA: IGI Global) 597–625. 10.4018/978-1-6684-3877-0.ch034

[B59] Pham L. Limbu Y. B. Bui T. K. Nguyen H. T. Pham H. T. (2019). Does e-learning service quality influence e-learning student satisfaction and loyalty? Evidence from Vietnam. Int. J. Educ. Technol. High. Educ. 16, 1–26. 10.1186/s41239-019-0136-3

[B60] Pham L. Williamson S. Berry R. (2018). Student perceptions of e-learning service quality, e-satisfaction, and e-loyalty. Int. J. Enterp. Inf. Syst. (IJEIS) 14, 19–40. 10.4018/IJEIS.2018070102

[B61] Rajasekaran V. A. Kumar K. R. Susi S. Mohan Y. C. Raju M. Hssain M. W. (2022). An evaluation of e-learning and user satisfaction. Int. J. Web-Based Learn. Teach. Technol. (IJWLTT) 17, 1–11. 10.4018/IJWLTT.20220301.oa3

[B62] Raymond R. (2015). When word-of-mouth goes online: evaluating the characteristics and effects of E-WOM communication. Int. J. Arts Sci. 8, 499–508.

[B63] Ringle C. Da Silva D. Bido D. (2015). Structural equation modeling with the SmartPLS. Braz. J. Market. 13, 56–73. 10.5585/remark.v13i2.2717

[B64] Rodríguez-Ardura I. Meseguer-Artola A. (2016). What leads people to keep on e-learning? An empirical analysis of users' experiences and their effects on continuance intention. Int. Learn. Environ. 24, 1030–1053. 10.1080/10494820.2014.926275

[B65] Saima F. N. Rahman M. H. A. Ghosh R. (2022). MFS usage intention during COVID-19 and beyond: an integration of health belief and expectation confirmation model. J. Econ. Adm. Sci. [Epub ahead of print]. 10.1108/JEAS-07-2021-0133

[B66] Sanford D. Ross D. Rosenbloom A. Singer D. (2017). Course convenience, perceived learning, and course satisfaction across course formats. E-J. Bus. Educ. Scholarship Teach. 11, 69–84.

[B67] Sasongko D. T. Handayani P. W. Satria R. (2022). Analysis of factors affecting continuance use intention of the electronic money application in Indonesia. Proc. Comput. Sci. 197, 42–50. 10.1016/j.procs.2021.12.116

[B68] Sharif S. P. Nia H. S. (2018). Structural Equation Modeling with AMOS. Tehran: Artin Teb.

[B69] Shavitt S. Cho H. (2016). Culture and consumer behavior: the role of horizontal and vertical cultural factors. Current opinion in psychology 8, 149–154. 10.1016/j.copsyc.2015.11.007 28083559 PMC5222543

[B70] She L. Ma L. Jan A. Sharif Nia H. Rahmatpour P. (2021a). Online learning satisfaction during COVID-19 pandemic among Chinese university students: the serial mediation model. Front. Psychol. 12:743936. 10.3389/fpsyg.2021.743936 34675851 PMC8524086

[B71] She L. Rasiah R. Turner J. J. Guptan V. Sharif Nia H. (2022). Psychological beliefs and financial well-being among working adults: the mediating role of financial behavior. Int. J. Soc. Econ. 49, 190–209. 10.1108/IJSE-07-2021-0389

[B72] She L. Sharif S. P. Nia H. S. (2021b). Psychometric evaluation of the chinese version of the modified online compulsive buying scale among Chinese young consumers. J. Asia-Pac. Bus. 22, 121–133. 10.1080/10599231.2021.1905493

[B73] Shehzadi S. Nisar Q. A. Hussain M. S. Basheer M. F. Hameed W. U. Chaudhry N. I. (2020). The role of digital learning toward students' satisfaction and university brand image at educational institutes of Pakistan: a post-effect of COVID-19. Asian Educ. Dev. Stud. 10, 276–294. 10.1108/AEDS-04-2020-0063

[B74] Siddiquei N. L. Khalid R. (2022). The learning style preferences of elearners in Pakistan. Int. J. Knowl Learn. 15, 49–66. 10.1504/IJKL.2022.11991629736072

[B75] Sohu (2020). How Many Hidden Pits are There in the 9.9-Yuan Python Class? Available online at: https://www.sohu.com/a/434011514_115865 (accessed September 22, 2021).

[B76] Son B. K. (2019). “Integrated e-learning paradigm in the twenty-first century: management education.” in Learning Technologies for Transforming Large-Scale Teaching, Learning, and Assessment, eds Sampson D. Spector J. M. Ifenthaler D. Isaías P. Sergis S. (Cham: Springer), 35–51. 10.1007/978-3-030-15130-0_3

[B77] Srinath K. R. (2017). Python—the fastest growing programming language. Int. Res. J. Eng. Technol. (IRJET) 4, 354–357.

[B78] Stevens C. J. Horrigan J. Heale R. Koren I. (2020). Northeastern Ontario nurses' perceptions of e-learning: an interpretive description. Nurse Educ. Today 92, 104509. 10.1016/j.nedt.2020.104509 32599472

[B79] Sugandini D. Istanto Y. (2022). E-learning system success adoption in Indonesia higher education. Acad. J. Interdiscip. Stud. 11, 149–158. 10.36941/ajis-2022-0013

[B80] Sulaymani O. Pratama A. R. Alshaikh M. Alammary A. (2022). The effects of previous experience and self efficacy on the acceptance of e-learning platforms among younger students in Saudi Arabia. Contemp. Educ. Technol. 14, ep349. 10.30935/cedtech/11524

[B81] Sulthana A. N. Vasantha S. (2019). Influence of electronic word of mouth e-WOM on purchase intention. Int. J. Sci. Technol. Res. 8, 1–5.

[B82] Suzianti A. Paramadini S. A. (2021). Continuance intention of E-learning: the condition and its connection with open innovation. J. Open Innov.: Technol. Mark. Complex. 17, 97. 10.3390/joitmc7010097

[B83] Thefuturesphere (2022). Python Elite Class: Object-Oriented Programming. Available online at: https://thefuturesphere.com/course/Python-professional (accessed *January* 26, 2022).

[B84] Tj H. W. Tanuraharjo H. H. (2020). The effect of online learning service quality on student satisfaction during COVID19 pandemic in 2020. J. Manaj. Indones. 20, 240. 10.25124/jmi.v20i3.3520

[B85] Truong N. N. Hieu V. M. Quoc T. H. A. (2017). An analysis of perceived student benefits in E-learning service case study on FPT University. J. Educ. Soc. Sci. 8, 2289–1552.

[B86] Tsimonis G. Dimitriadis S. (2014). Brand strategies in social media. Market. Intell. Plan. 32, 328–344. 10.1108/MIP-04-2013-0056

[B87] Twum K. K. Kosiba J. P. B. Hinson R. E. Gabrah A. Y. B. Assabil E. N. (2022). Determining mobile money service customer satisfaction and continuance usage through service quality. J. Financ. Serv. Market. [Epub ahead of print]. 10.1057/s41264-021-00138-5

[B88] Ung L. L. Labadin J. Mohamad F. S. (2022). Computational thinking for teachers: development of a localised E-learning system. Comput. Educ. 177, 104379. 10.1016/j.compedu.2021.104379

[B89] Yassine S. Khalifa M. Franck P. (2017). “Towards a multidimensional model to study a critical success factors affecting continuity and success in e-learning systems.” in 2017 10th International Conference on Developments in eSystems Engineering (DeSE) (Paris, France). 10.1109/DeSE.2017.26

[B90] Yoon C. Kim S. (2007). Convenience and TAM in a ubiquitous computing environment: the case of wireless LAN. Electron. Commer. Res. Appl. 6, 102–112. 10.1016/j.elerap.2006.06.009

[B91] Zhang Y. N. (2017). Python Salary has Gone Up Again! What Can I Do! Available online at: https://news.51cto.com/art/201711/559529.html (accessed November 21, 2021).

[B92] Zimmerman W. Altman B. Simunich B. Shattuck K. Burch B. (2020). Evaluating online course quality: a study on implementation of course quality standards. Online Learn. 24, 147–163. 10.24059/olj.v24i4.2325

